# Liposomal mitoxantrone, venetoclax and azacitidine in relapsed/refractory acute myeloid leukaemia and early induction failure: a single-centre real-world study

**DOI:** 10.3389/fonc.2026.1909165

**Published:** 2026-08-19

**Authors:** Jiaxin Zhao, Yanping Yang, Yiqi Dai, Sujun Gao, Yehui Tan

**Affiliations:** Department of Haematology, The First Hospital of Jilin University, Changchun, Jilin, China

**Keywords:** acute myeloid leukaemia, azacitidine, liposomal mitoxantrone, relapsed/refractory AML, venetoclax failure

## Abstract

**Introduction:**

Patients with relapsed/refractory acute myeloid leukaemia have poor outcomes, particularly after failure of venetoclax-based therapy.

**Methods:**

We retrospectively analysed 16 adults with relapsed/refractory AML or early induction failure who received liposomal mitoxantrone, venetoclax and azacitidine (MVA) at a single centre between March 2024 and August 2025. The primary endpoint was composite complete remission (CRc), defined as complete remission or complete remission with incomplete haematological recovery.

**Results:**

Median age was 58.5 years. Eight patients had relapsed AML, five had refractory AML and three had early induction failure; nine had previously failed venetoclax-based therapy. CRc was achieved in 10/16 patients (62.5%; 95% CI, 35.4% -84.8%), and the overall response rate was 14/16 (87.5%; 95% CI, 61.7% -98.4%). Among patients with prior venetoclax failure, 7/9 achieved CRc (77.8%; 95% CI, 40.0% -97.2%). Four patients proceeded to allogeneic haematopoietic stem cell transplantation (allo-HSCT). Grade 3 -4 neutropenia and thrombocytopenia occurred in 16/16 and 14/16 patients, respectively. No treatment-related deaths occurred during MVA therapy.

**Discussion:**

These findings suggest that MVA had clinical activity as a salvage regimen for difficult-to-treat AML, including patients with prior venetoclax failure, with expected myelosuppression and potential bridging utility before allo-HSCT. Prospective studies are needed to confirm efficacy and define patient selection.

## 1 Introduction

Acute myeloid leukaemia (AML) is the most common acute leukaemia in adults (1). Although 64%–85% of adult patients achieve complete remission (CR) with initial induction chemotherapy (2–6), disease relapse following treatment remains a major challenge. Approximately 50%–80% of patients ultimately progress to relapsed or refractory AML (R/R AML), which is associated with a five-year survival rate of less than 10% (3–7). These outcomes highlight the need for effective and tolerable salvage strategies for patients with R/R AML.

Currently, there is no universally standardised treatment for R/R AML, and clinical strategies are highly individualised. Haematopoietic stem cell transplantation (HSCT) remains the only potentially curative option for R/R AML (8); however, only a minority of patients are eligible for transplantation (9), and even among those who successfully undergo HSCT, the five-year overall survival rate remains between 30% and 50% (10–12). Conventional salvage chemotherapy can rapidly reduce tumour burden and create opportunities for subsequent transplantation, but it is associated with significant toxicity and is therefore suitable only for patients who can tolerate intensive therapy. Venetoclax plus azacitidine has improved outcomes in patients with AML who are unfit for intensive chemotherapy (7, 13); however, primary or acquired resistance remains common and is associated with poor subsequent outcomes (14, 15). After failure of venetoclax-based therapy, available salvage options are limited, and reported complete remission rates remain modest (16, 17). Therefore, overcoming venetoclax resistance has emerged as a pivotal challenge in the management of R/R AML.

Recent preclinical studies suggest that venetoclax resistance may be associated with altered mitochondrial calcium metabolism in leukaemia stem cells (LSCs) (18, 19). Mitoxantrone, an anthracenedione compound, has been identified in preclinical studies as a pharmacologic modulator of mitochondrial calcium homeostasis, including MCU-related pathways, with activity against resistant LSCs (18). Compared with conventional anthracyclines, mitoxantrone exhibits enhanced antileukaemic activity and reduced cardiotoxicity (20, 21). Liposomal mitoxantrone (Lipo-MIT) was developed to improve drug delivery and pharmacokinetic properties, with the aim of enhancing antitumour activity while reducing systemic toxicity, including cardiotoxicity (22). Multiple clinical studies have demonstrated the efficacy of Lipo-MIT-containing regimens in acute leukaemia (23). However, real-world data on the clinical activity and safety of MVA remain limited, particularly in patients with R/R AML who have failed venetoclax plus azacitidine-based therapy or harbour high-risk genetic abnormalities. To address this gap, we conducted a single-centre retrospective real-world study to describe the clinical activity and safety of MVA in patients with R/R AML or early induction failure.

## 2 Materials and methods

### 2.1 Study design and patients

This single-centre retrospective cohort study included consecutive adult patients who received liposomal mitoxantrone, venetoclax and azacitidine (MVA) at The First Hospital of Jilin University between March 2024 and August 2025. The data cut-off date was 1 December 2025. Patients were diagnosed with AML according to the 2016 World Health Organization classification of myeloid neoplasms and acute leukaemia (24).

Eligible patients were adults aged ≥18 years with confirmed non-acute promyelocytic AML who met one of the following disease criteria: (i) R/R AML, defined as failure to achieve CR, CRh or CRi after at least two cycles of standard induction therapy, or relapse after a prior remission, including reappearance of peripheral blood blasts, bone marrow blasts ≥5% after exclusion of other causes, or extramedullary involvement; or (ii) early induction failure, defined as persistent bone marrow blasts >10% after one cycle of chemotherapy with or without targeted therapy, or progressive peripheral blasts after initial treatment. Early induction failure was analysed separately from classical R/R AML because these patients had not fulfilled conventional refractory criteria after two induction cycles. These patients were included because early salvage therapy had been selected by the treating physicians in routine practice when the likelihood of benefit from a second conventional induction cycle was considered low.

Additional inclusion requirements included ECOG performance status 0–2, life expectancy ≥3 months, available efficacy data post-treatment, and adequate organ function. Exclusion criteria comprised uncontrolled systemic illnesses, concurrent malignancies, or any condition precluding study participation. Patients with clinically controlled infections receiving appropriate anti-infective therapy were eligible, whereas uncontrolled or life-threatening infections precluding chemotherapy were excluded. The study protocol adhered to the principles of the Declaration of Helsinki and Good Clinical Practice guidelines. The requirement for informed consent was waived due to the retrospective nature of the study.

Prior venetoclax failure was defined as primary non-response to a venetoclax-based regimen after at least one evaluable treatment cycle, or relapse/progression after a previous response to venetoclax-based therapy. Patients who discontinued venetoclax for reasons unrelated to lack of efficacy, relapse or progression were not classified as having venetoclax failure. ELN 2022 risk was assigned using pre-MVA cytogenetic and molecular data. Karyotypes were classified as normal, abnormal non-complex or complex, with complex karyotype defined as ≥3 clonal chromosomal abnormalities. Prior treatment lines were defined as distinct systemic anti-leukaemic strategies; repeated cycles or consolidation within the same strategy were counted as one line, whereas treatment changes for non-response, relapse, progression or molecular/MRD recurrence were counted as new lines. Prior treatment intensity was classified according to any exposure to intensive induction, consolidation, re-induction or salvage therapy; patients without such exposure were classified as having received lower-intensity therapy only. Prior intensive salvage therapy was reported separately.

### 2.2 Treatment

All patients received MVA, consisting of liposomal mitoxantrone, venetoclax and azacitidine. The dosing schedule and dose-adjustment approach were as follows: Liposomal mitoxantrone was administered intravenously at a dose of 24 mg/m² on day 1. Seven patients not receiving a strong CYP3A4 inhibitor received venetoclax 100 mg on day 1, 200 mg on day 2 and 400 mg on days 3–14. Nine patients receiving concomitant posaconazole received venetoclax 100 mg once daily on days 1–14. All patients completed the planned 14-day venetoclax course without interruption, early discontinuation or dose reduction beyond the prespecified adjustment for concomitant posaconazole. Azacitidine was administered at a dose of 75 mg/m²/day either subcutaneously or intravenously for seven consecutive days (days 1–7). Response was assessed after one cycle of MVA unless clinically indicated earlier. Additional cycles, dose modifications or transition to allo-HSCT were determined by the treating physicians according to response, toxicity and transplant availability. Supportive care, including antimicrobial prophylaxis, tumour lysis syndrome prophylaxis, transfusion support and haematopoietic growth factor use, was provided according to institutional practice at the discretion of the treating physicians. Prior anthracycline and anthracenedione exposure was reviewed using actual delivered doses normalised to body surface area, where available. Doxorubicin-equivalent doses were calculated using conversion factors of 5.0 for idarubicin and 0.5 for daunorubicin. Aclarubicin and liposomal mitoxantrone were reported separately because validated conversion factors were unavailable. Post-MVA treatment was classified as an additional MVA cycle, non-MVA consolidation, other lower-intensity systemic therapy, direct allo-HSCT, or no further anti-leukaemic therapy. Among responders who received subsequent anti-leukaemic treatment or proceeded to allo-HSCT, the interval to the next intervention was calculated from MVA initiation to the start of the first subsequent treatment or allo-HSCT. Treatment delay was defined as failure to initiate planned subsequent systemic anti-leukaemic therapy within 28 days after MVA initiation. Patients proceeding directly to allo-HSCT or declining further treatment for personal reasons were excluded from this analysis.

### 2.3 Outcomes and assessment

The primary endpoint was the composite complete remission (CRc) rate, defined as the proportion of patients achieving complete remission (CR) or CR with incomplete haematological recovery (CRi) as best overall response. Secondary endpoints included overall response rate (ORR), event-free survival (EFS), overall survival (OS), measurable residual disease (MRD) negativity and safety. ORR was defined as CR, CRi, morphologic leukaemia-free state (MLFS) or partial remission (PR) as best overall response. EFS was defined as the time from MVA initiation to treatment failure, relapse after response or death from any cause, whichever occurred first. For patients with no response to MVA, treatment failure was dated at the first response assessment. OS was defined as the time from MVA initiation to death from any cause. Treatment response was assessed according to the European LeukaemiaNet 2022 recommendations.

MRD was assessed by multiparameter flow cytometry at response assessment. MRD negativity was defined as <0.1% leukaemia-associated immunophenotype-positive cells among CD45-positive white blood cells. MRD status at the first response assessment was reported as part of the initial treatment-response evaluation. Because subsequent assessments were performed at non-uniform intervals, longitudinal MRD trajectories were categorised descriptively as sustained negativity, conversion from positivity to negativity, persistent positivity or MRD re-emergence. Associations between MRD status at the first response assessment and subsequent relapse, death and post-response OS were explored descriptively.

Safety was evaluated according to CTCAE version 5.0. Treatment-emergent adverse events observed during MVA treatment were recorded. Treatment-emergent infections were defined as infections that newly occurred or showed clinically meaningful worsening after MVA initiation; controlled pre-existing infections without documented worsening were recorded as baseline conditions. Infections were classified as microbiologically documented when a clinically relevant organism was identified together with compatible clinical or radiological findings, or as clinically documented when a compatible infectious focus was present without an identified pathogen. Haematological recovery was assessed from MVA initiation. ANC recovery was defined as the first documented ANC ≥0.5 × 10^9^/L, and platelet recovery as the first platelet count ≥50 × 10^9^/L. Patients without documented recovery before death or last follow-up were classified as not recovered. The durations of ANC <0.5 × 10^9^/L and platelet count <50 × 10^9^/L, cumulative MVA-related hospitalisation, infectious events, ICU admission, infection-related mortality, and pre- and post-MVA LVEF were recorded. Hospitalisation duration was calculated as the cumulative number of inpatient days during the MVA-related treatment period, including short-term readmissions for cytopenia management. LVEF was assessed by transthoracic echocardiography before MVA initiation and after treatment when clinically feasible.

### 2.4 Statistical analysis

All statistical analyses were performed using IBM SPSS Statistics version 27.0 (IBM Corp., Armonk, NY, USA). Categorical variables are presented as frequencies and percentages, and continuous variables as medians with ranges. CRc and ORR were reported with two-sided 95% exact binomial confidence intervals (CIs). OS and EFS were estimated using the Kaplan–Meier method, and median follow-up was estimated using the reverse Kaplan–Meier method. Among responders, an exploratory post-response OS analysis was performed with the time origin reset to each patient’s first response assessment and stratified by MRD status at that assessment. Median post-response OS and its 95% CI were estimated using the Kaplan–Meier method, with confidence intervals for median survival obtained from pointwise confidence limits based on the complementary log–log transformation with Greenwood variance estimates. Survival distributions were compared using the log-rank test.

## 3 Results

### 3.1 Patient baseline characteristics

**Figure 1:**
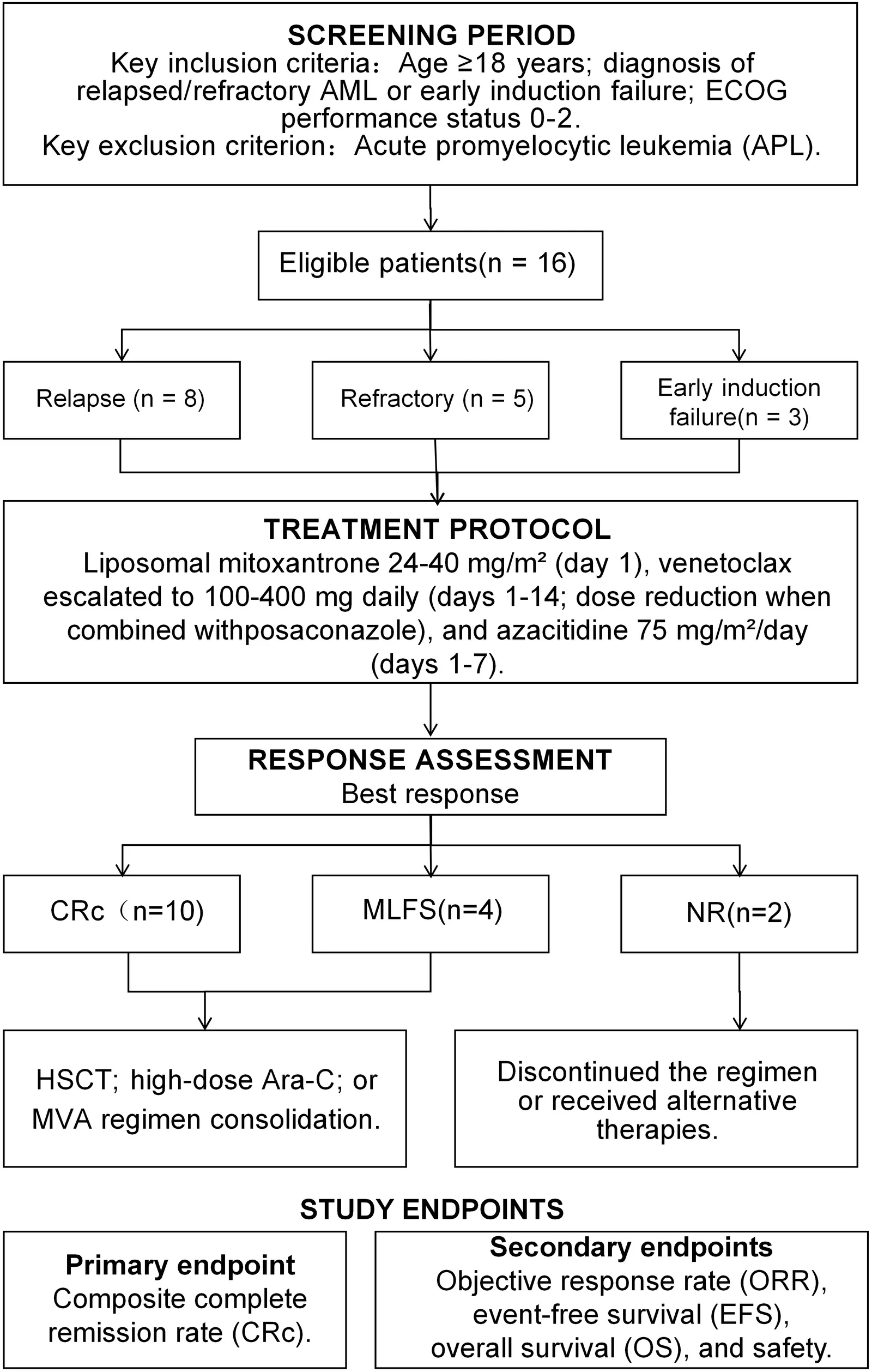
Study flow diagram. AML, acute myeloid leukaemia; Lipo-MIT, liposomal mitoxantrone; MVA, liposomal mitoxantrone, venetoclax and azacitidine.

Sixteen patients were included in both the efficacy and safety analyses, as shown in the study flow diagram (**Figure 1**). The median age was 58.5 years (range, 20–73 years). Eight patients had relapsed AML, five had refractory AML and three had early induction failure. Baseline clinical, cytogenetic and molecular characteristics, including ELN 2022 risk, baseline bone marrow and peripheral blood blast percentages, first remission duration among patients with a documented prior remission (n = 10), ECOG performance status, and relevant comorbidities and medical history, are summarised in **Table 1**. Three patients (18.8%) had relevant comorbidities or medical histories: one had diabetes mellitus, one had a history of thyroid nodule surgery and one had a history of hepatitis B virus infection managed with entecavir. Selected patient-level disease, genetic, prior-treatment, response and follow-up characteristics are provided in **Supplementary Table 1**. The median number of detected mutated genes per patient was 2.5 (range, 0–5), and nine patients (56.3%) harboured at least two co-occurring mutated genes. TP53 and BCOR mutations were not detected, whereas NPM1 mutations were identified in two patients.

**Table 1:** Baseline clinical characteristics of the study cohort.

Clinical features	Value
Median age	58.5 (20–73)
*Gender, n (%)*	
Male	7 (43.8%)
Female	9 (56.3%)
*AML type, n (%)*	
De novo AML	13 (81.3%)
Secondary AML (transformed from MDS/CMML)	3 (18.8%)
*ELN 2022 risk classification, n (%)*	
Favourable	1 (6.3%)
Intermediate	5 (31.3%)
Adverse	10 (62.5%)
*Disease status before MVA*	
Refractory	5 (31.3%)
Relapse	8 (50.0%)
Early Induction Failure	3 (18.8%)
*Molecular mutations, n (%)*	
FLT3(FLT3-TKD/FLT3-ITD)	4 (25.0%)
ASXL1	6 (37.5%)
IDH2	2 (12.5%)
U2AF1	3 (18.8%)
CEBPA	2 (12.5%)
RUNX1	2 (12.5%)
RAS(KRAS/NRAS)	4 (25.0%)
TP53	0 (0.0%)
NPM1	2 (12.5%)
BCOR	0 (0.0%)
*Chromosomal abnormalities, n (%)*	
Normal karyotype	7 (43.8%)
Abnormal non-complex karyotype	7 (43.8%)
Complex karyotype	2 (12.5%)
*Baseline disease burden*	
ECOG 0–1, n (%)	7 (43.8%)
ECOG 2, n (%)	9 (56.3%)
Bone marrow blasts at baseline, %, median (range)	19 (5–85)
Peripheral blood blasts at baseline, %, median (range)	3 (0–52)
*Relevant comorbidities and medical history, n (%)*	
At least one relevant comorbidity or medical history	3 (18.8%)
Diabetes mellitus	1 (6.3%)
History of thyroid nodule surgery	1 (6.3%)
History of hepatitis B virus infection receiving entecavir	1 (6.3%)
No documented relevant comorbidity or medical history	13 (81.3%)
*Prior treatment history*	
Number of prior treatment lines, median (range)	2 (1–4)
First remission duration (n = 10), months, median (range)	7.5 (3–20)
Prior venetoclax failure, n (%)	9 (56.3%)
Duration of prior venetoclax exposure, months, median (range)	1.9 (0.2–3.7)
*Pattern of venetoclax failure, n (%)*	
Primary failure	2 (22.2%)
Secondary failure after a prior response	7 (77.8%)
Interval from venetoclax discontinuation to MVA initiation, days, median (range)	35 (10–150)
Prior intensive salvage therapy, n (%)	8 (50.0%)

Prior VEN exposure was calculated from actual treatment days (30 days = 1 month). Primary failure was defined as NR after ≥1 evaluable VEN-based cycle and secondary failure as relapse/progression after a prior response. Intensive salvage regimens were HA (n = 5), IA (n = 3), HAA (n = 1) and MA (n = 1), with overlapping categories. Mutation counts refer to mutated genes and exclude cytogenetic abnormalities and fusion genes; additional mutations are listed in **Suppelementary Table 1**. First remission duration was calculated from CR/CRi/CRh to haematological relapse (n = 10).

All patients had received prior systemic anti-leukaemic therapy, with a median of 2 prior treatment lines (range, 1–4). Eleven patients (68.8%) had prior conventional anthracycline exposure, including three who had additionally received liposomal mitoxantrone. The median cumulative doxorubicin-equivalent dose was 180 mg/m² (range, 90–360 mg/m²). Because no validated doxorubicin-equivalent conversion factor was available, prior liposomal mitoxantrone exposure was reported separately; the three patients achieved CR, CRi and NR, respectively, with the current MVA regimen. Prior anthracycline or anthracenedione exposure did not lead to a reduction in the liposomal mitoxantrone dose, and all patients received 24 mg/m² on day 1. Nine patients (56.3%) had failed prior venetoclax-based therapy, comprising two patients with primary failure and seven with secondary failure after a previous response. The median cumulative duration of prior venetoclax exposure was 1.9 months (range, 0.2–3.7 months); the shortest (0.2 months) was a complete, planned 7-day venetoclax course within a cytarabine-based combination, not premature discontinuation, calculated from the actual number of treatment days. The median interval from venetoclax discontinuation to MVA initiation was 35 days (range, 10–150 days). Eight patients (50.0%) had received at least one prior intensive salvage regimen, including homoharringtonine plus cytarabine (HA) in five patients, idarubicin plus cytarabine (IA) in three, homoharringtonine, cytarabine plus aclarubicin (HAA) in one and liposomal mitoxantrone plus cytarabine (MA) in one; regimen categories were not mutually exclusive. These aggregate prior-treatment characteristics are summarised in **Table 1**.

### 3.2 Treatment response

Among the 16 patients treated with the MVA regimen, the best overall response assessment was as follows: 4 patients (25.0%) achieved complete remission (CR), 6 patients (37.5%) achieved CR with incomplete haematological recovery (CRi), 4 patients (25.0%) achieved morphologic leukaemia-free state (MLFS), and 2 patients (12.5%) had non-response (NR). The overall CRc rate was 62.5% (10/16; 95% CI, 35.4%–84.8%), and the ORR was 87.5% (14/16; 95% CI, 61.7%–98.4%). No patient achieved PR; therefore, ORR comprised CR, CRi and MLFS in this cohort. In a sensitivity analysis excluding the three patients with early induction failure, the CRc and ORR rates among patients meeting conventional R/R AML criteria were 8/13 (61.5%; 95% CI, 31.6%–86.1%) and 11/13 (84.6%; 95% CI, 54.6%–98.1%), respectively.

Among the nine patients with prior venetoclax failure, 7 achieved CRc (77.8%; 95% CI, 40.0%–97.2%), including patients with primary non-response to venetoclax-based therapy and those who relapsed after a previous venetoclax-associated response.

Response was also explored according to prior treatment intensity. Eleven patients had received at least one intensive induction, re-induction, consolidation or salvage regimen before MVA. Among these patients, 6 achieved CRc (54.5%; 95% CI, 23.4%–83.3%) and 10 achieved ORR (90.9%; 95% CI, 58.7%–99.8%). Among the five patients who had received lower-intensity therapy only, CRc and ORR were each achieved in 4 patients (80.0%; 95% CI, 28.4%–99.5%). These findings were interpreted descriptively because of the small and imbalanced subgroups, heterogeneous prior regimens and substantial confounding by disease status and prior venetoclax exposure.

Response rates varied according to disease status before MVA. CRc was achieved in 4/5 refractory patients (80.0%; 95% CI, 28.4%–99.5%), 2/3 patients with early induction failure (66.7%; 95% CI, 9.4%–99.2%) and 4/8 relapsed patients (50.0%; 95% CI, 15.7%–84.3%). ORR was 5/5 (100.0%; 95% CI, 47.8%–100.0%), 3/3 (100.0%; 95% CI, 29.2%–100.0%) and 6/8 (75.0%; 95% CI, 34.9%–96.8%), respectively.

By ELN 2022 risk, CRc was achieved in 0/1 favourable-risk patient (0%; 95% CI, 0%–97.5%), 2/5 intermediate-risk patients (40.0%; 95% CI, 5.3%–85.3%) and 8/10 adverse-risk patients (80.0%; 95% CI, 44.4%–97.5%). The corresponding ORR rates were 1/1 (100.0%; 95% CI, 2.5%–100.0%), 3/5 (60.0%; 95% CI, 14.7%–94.7%) and 10/10 (100.0%; 95% CI, 69.2%–100.0%), respectively.

Responses were also observed in several molecularly defined subgroups, although patient numbers were very small. CRc was achieved in 5/6 patients with ASXL1 mutations (83.3%; 95% CI, 35.9%–99.6%) and 2/4 patients with RAS pathway mutations (50.0%; 95% CI, 6.8%–93.2%). Both patients with complex karyotype achieved CRc (100.0%; 95% CI, 15.8%–100.0%). These observations are exploratory and were not powered for subgroup inference.

At the first response assessment, 7 of 14 responders were MRD-negative (50.0%; 95% CI, 23.0%–77.0%), including 6 of 10 patients achieving CRc (60.0%; 95% CI, 26.2%–87.8%). MRD negativity at the first assessment was observed in 2/6 ASXL1-mutated responders, 1/4 responders with NRAS/KRAS mutations and 1/3 FLT3-mutated responders, but in neither of the two responders with complex karyotype.

### 3.3 Safety

All 16 patients were included in the safety analysis. Treatment-emergent adverse events observed during MVA treatment were graded according to CTCAE version 5.0. Haematological toxicities were the most common and prominent, with the incidence of grade 3–4 adverse events detailed in **Table 2**. Grade 3–4 neutropenia occurred in all 16 patients (100.0%). Febrile neutropenia occurred in 13 patients (81.3%). The incidences of grade 3–4 thrombocytopenia and anaemia were 87.5% (14/16) and 75.0% (12/16), respectively. ANC fell below 0.5 × 10^9^/L in 15 patients, all of whom subsequently recovered. The median duration below this threshold was 29 days (range, 8–115 days), and the median time from MVA initiation to recovery was 34 days (range, 25–115 days). Platelet counts fell below 50 × 10^9^/L in 14 patients; 13 recovered, whereas one died before recovery. Among those who recovered, the median time from MVA initiation to platelet recovery was 38 days (range, 18–119 days), and the median duration below 50 × 10^9^/L was 24 days (range, 8–119 days).

**Table 2:** Treatment-emergent adverse events.

Adverse events	Any grade	Grade 1–2	Grade 3–4
*Haematological toxicity, no. (%)*			
Leukopenia	16 (100.0)	2 (12.5)	14 (87.5)
Thrombocytopenia	16 (100.0)	2 (12.5)	14 (87.5)
Neutropenia	16 (100.0)	0 (0.0)	16 (100.0)
Anaemia	16 (100.0)	4 (25.0)	12 (75.0)
Febrile neutropenia	13 (81.3)	0 (0.0)	13 (81.3)
*Non-haematological toxicity, no. (%)*			
Hypokalaemia	7 (43.8)	7 (43.8)	0 (0.0)
Hyponatraemia	5 (31.3)	5 (31.3)	0 (0.0)
Perianal infection	2 (12.5)	2 (12.5)	0 (0.0)
ALT increased	2 (12.5)	2 (12.5)	0 (0.0)
AST increased	2 (12.5)	2 (12.5)	0 (0.0)
Pulmonary infection	3 (18.8)	2 (12.5)	1 (6.3)
Fatigue	3 (18.8)	3 (18.8)	0 (0.0)
ALP increased	2 (12.5)	2 (12.5)	0 (0.0)
Oral infection	1 (6.3)	1 (6.3)	0 (0.0)
Sepsis	1 (6.3)	0 (0.0)	1 (6.3)

Infection categories were not mutually exclusive; one patient could contribute to more than one infection category.

At MVA initiation, 13 patients (81.3%) had clinically controlled pre-existing infections. Six patients (37.5%) developed new or clinically worsening infections after MVA initiation, including pulmonary infections in three patients (18.8%), perianal infections in two (12.5%) and an oral infection in one (6.3%). Five of the six infections were microbiologically documented, comprising bacterial infections in three patients and fungal infections in two. One patient with Pseudomonas aeruginosa sepsis also tested positive for SARS-CoV-2. One additional patient had a clinically documented perianal infection without an identified pathogen. A respiratory isolate of Candida glabrata in another patient was considered colonisation and was not classified as a causative fungal infection. No patient required ICU admission, and no infection-related deaths occurred. The median cumulative hospitalisation duration related to MVA treatment was 30.5 days (range, 20–65 days). Among the 12 responders who received a subsequent anti-leukaemic intervention or proceeded directly to allo-HSCT, the median interval from MVA initiation to the next intervention was 56.5 days (range, 35–131 days). Among the 11 responders for whom subsequent systemic anti-leukaemic therapy was planned, 10 initiated treatment after the planned 28-day interval, whereas one was unable to initiate further therapy because of delayed haematological recovery.

Hypokalaemia and hyponatraemia occurred in seven (43.8%) and five (31.3%) patients, respectively, and were all grade 1–2. Toxicities were managed with supportive care, including blood product transfusion, haematopoietic growth factors and anti-infective therapy. No treatment-related deaths occurred during MVA therapy, and no deaths occurred within 30 or 60 days of MVA initiation. Baseline LVEF was available for all 16 patients and post-MVA LVEF for 15. Among the 15 patients with paired assessments, the median change was +1 percentage point (range, −7 to +7), with no symptomatic heart failure or other treatment-emergent cardiac events.

### 3.4 Post-MVA treatment

**Figure 2:**
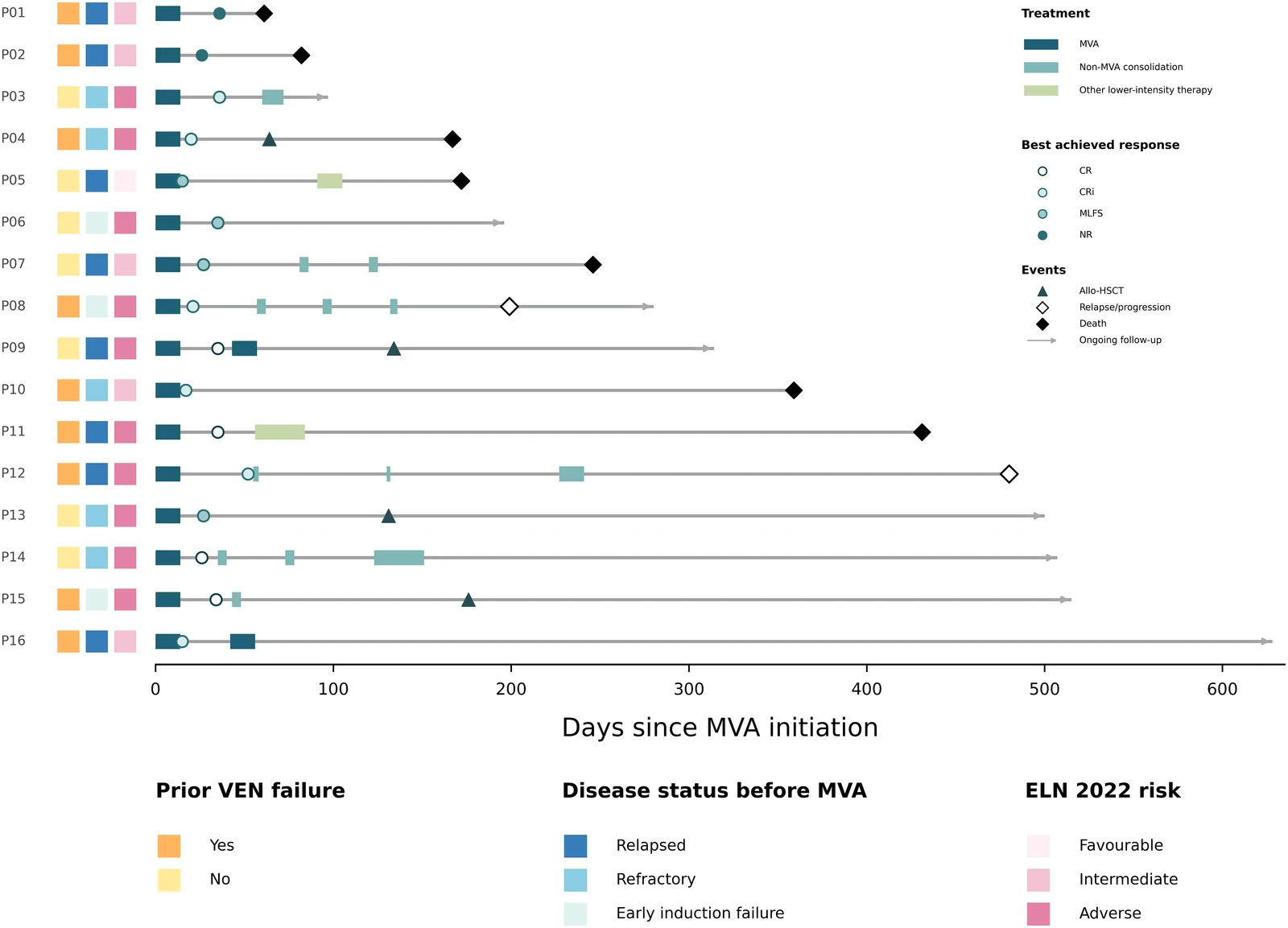
Patient-level swimmer plot showing treatment courses and outcomes after MVA initiation. The plot displays best response, subsequent anti-leukaemic therapy, allo-HSCT, relapse, death and ongoing follow-up. Time is shown in days from MVA initiation.

Among the 14 responders, two received an additional MVA cycle, six received non-MVA consolidation, two received other lower-intensity systemic therapy, two proceeded directly to allo-HSCT, and two received no further anti-leukaemic therapy. Two additional responders subsequently underwent allo-HSCT after post-MVA therapy, resulting in four transplants overall. Both patients with NR received no further anti-leukaemic therapy. Individual treatment courses, responses, subsequent therapy, transplantation, relapse, death and ongoing follow-up are shown in **Figure 2**.

Serial MRD data were available for 13 of the 14 responders. Four patients remained MRD-negative, four converted from MRD positivity to negativity, three remained MRD-positive and two showed MRD re-emergence; one responder had no subsequent MRD assessment. During subsequent follow-up, MRD clearance occurred in 2/4, 2/3, 2/2 and 2/2 initially MRD-positive patients in the ASXL1-mutated, NRAS/KRAS-mutated, FLT3-mutated and complex-karyotype subgroups, respectively. These longitudinal findings were interpreted descriptively because MRD assessments were performed at non-uniform intervals.

### 3.5 Survival and transplantation outcomes

**Figure 3:**
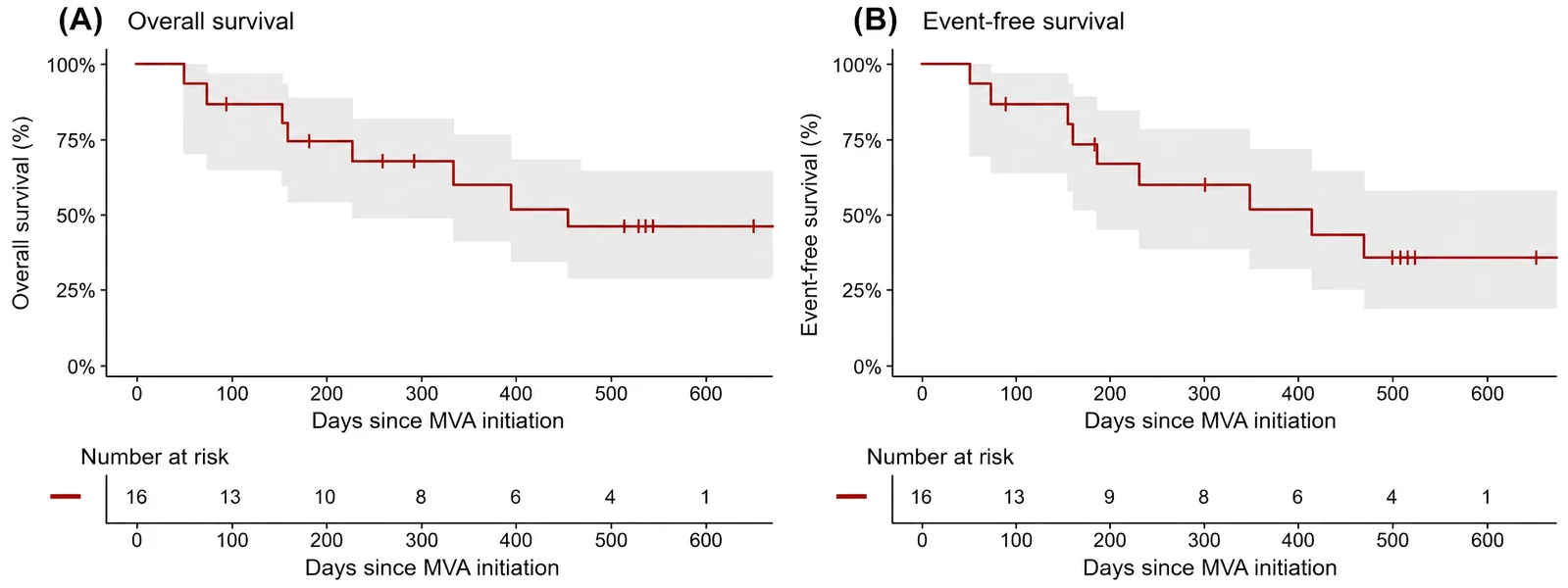
Kaplan–Meier estimates of overall survival and event-free survival. **(A)** Overall survival in the entire cohort. Median OS was 431 days (95% CI, 172 days–NE), with an estimated 1-year OS rate of 57.1% (95% CI, 27.3%–78.6%). **(B)** Event-free survival in the entire cohort. Median EFS was 431 days (14.2 months; 95% CI, 172 days–NE), with an estimated 1-year EFS rate of 50.8% (95% CI, 23.0%–73.2%). Tick marks indicate censored observations. Numbers at risk are shown below each panel. NE, not estimable.

At data cut-off, 7 deaths and 9 EFS events had occurred. The nine EFS events comprised two treatment failures at the first response assessment, two relapses after an objective response, and five deaths without a preceding treatment-failure or relapse event. The median follow-up estimated using the reverse Kaplan–Meier method was 500 days (95% CI, 280–515 days), corresponding to 16.4 months (95% CI, 9.2–16.9 months). The median OS was 431 days (95% CI, 172 days–NE), with an estimated 1-year OS rate of 57.1% (95% CI, 27.3%–78.6%). The median EFS was 431 days (14.2 months; 95% CI, 172 days–NE), with an estimated 1-year EFS rate of 50.8% (95% CI, 23.0%–73.2%) (**Figure 3**). These survival estimates should be interpreted descriptively because of the small cohort size and limited number of events.

Four patients subsequently proceeded to allo-HSCT after MVA treatment. Prior to transplantation, two patients were in CR, one in CRi and one in MLFS; three patients were MRD-negative and one was MRD-positive. Three patients were alive without documented relapse at the final follow-up. The fourth patient, who underwent transplantation in CRi, died from severe transplant-related complications on post-transplant day 103 without preceding relapse and was classified as non-relapse mortality. These post-transplant outcomes are descriptive only.

All four patients with sustained MRD negativity were alive without relapse. Among patients who converted from MRD positivity to negativity, two remained alive without relapse and two died without preceding relapse. Persistent MRD positivity was followed by two deaths and one relapse, whereas one of the two patients with MRD re-emergence subsequently relapsed. One death and one relapse occurred among the seven initially MRD-negative responders, compared with four deaths and one relapse among the seven initially MRD-positive responders. In the exploratory post-response OS analysis, median post-response OS was not reached in the MRD-negative group (95% CI, 342 days–NE) and was 396 days in the MRD-positive group (95% CI, 147 days–NE); the difference was not statistically significant (log-rank P = 0.17).

## 4 Discussion

In this single-centre retrospective cohort, MVA achieved a CRc rate of 62.5% and an ORR of 87.5%; notably, 7 of 9 patients with prior venetoclax failure achieved CRc. These results indicate an efficacy signal in a difficult-to-treat and heterogeneous population but should not be interpreted as comparative evidence of superiority. Myelosuppression was frequent but generally reversible, with median times to ANC and platelet recovery of 34 and 38 days, respectively. The 30- and 60-day mortality rates were both 0%, and no ICU admission or infection-related death occurred. Prior anthracycline or anthracenedione exposure did not result in reduction of the liposomal mitoxantrone dose, and paired echocardiographic assessments showed no clinically meaningful median decline in LVEF. Nevertheless, the small cohort and incomplete cumulative-dose equivalence for some prior agents preclude firm conclusions regarding cardiotoxicity.

Sustained MRD negativity was accompanied by favourable survival and relapse outcomes, whereas persistent or re-emergent MRD was more frequently followed by death or relapse. However, the small number of patients and events precludes firm conclusions regarding the prognostic value of MRD or differences across genetic subgroups.

In this study, responses to MVA were also observed in patients harbouring adverse cytogenetic or molecular features. Both patients with complex karyotype achieved CRc. One patient with highly adverse cytogenetic features, including inv(3) and monosomy 7, achieved CR following MVA treatment and remained in remission for approximately 6 months before proceeding to allo-HSCT, and was still relapse-free at the last follow-up. Previous studies have reported limited responses to conventional chemotherapy in complex-karyotype AML, with CR rates generally ranging from 25% to 50%, highlighting this subgroup as a persistent therapeutic challenge in R/R AML (25–27). Among the six patients with ASXL1 mutations, five achieved CRc, including two patients who had previously failed venetoclax plus azacitidine-based therapy. ASXL1 mutations have been recognised as an independent adverse prognostic factor in AML and are generally associated with inferior treatment outcomes (28–32). These responses suggest that MVA may retain antileukaemic activity in a subset of patients with epigenetic dysregulation or secondary-like AML biology. In contrast, responses among patients with FLT3-ITD mutations were heterogeneous: one of two patients achieved CRi, whereas the other had no response. The non-responding patient harboured concurrent FLT3-ITD and NPM1 mutations and had experienced early relapse after achieving remission with a prior venetoclax/azacitidine regimen. In the R/R AML or venetoclax-exposed setting, FLT3-associated kinase pathway activation, enhanced MAPK signalling and MCL-1-dependent anti-apoptotic rewiring may contribute to treatment resistance (31, 33–36). Overall, complex karyotype, ASXL1 mutation and FLT3-ITD represent distinct layers of high-risk AML biology. The responses observed in this study suggest that MVA may retain antileukaemic activity in selected patients with high-risk genetic backgrounds; however, given the small sample size, these subgroup findings should be interpreted as exploratory and hypothesis-generating.

Previous studies have implicated RAS/MAPK pathway activation in venetoclax resistance in AML (15, 33, 37, 38). In this cohort, CR/CRi was achieved in 2 of 4 patients with RAS pathway mutations, including both patients who had failed prior venetoclax-based therapy. This exploratory finding is consistent with the hypothesis that liposomal mitoxantrone may retain activity in a subset of RAS-mutated AML through mechanisms complementary to venetoclax/azacitidine, including topoisomerase II inhibition, DNA damage induction and effects on leukaemia stem-cell metabolic adaptation (33, 37–40). However, the small number of RAS-mutated patients precludes conclusions regarding the ability of MVA to overcome RAS-associated venetoclax resistance. Larger prospective studies with molecular response assessment are warranted.

For patients with relapsed or refractory AML, allo-HSCT remains the only potentially curative treatment for many patients, and effective bridging therapy is essential to reduce disease burden and enable successful transplantation (7, 41–43). In the present study, 4 patients proceeded to allo-HSCT following MVA treatment. Among them, 2 proceeded directly to allo-HSCT after MVA and 2 after further therapy (an additional MVA cycle in one and high-dose cytarabine consolidation in the other). The three patients transplanted in CR or MLFS remain in sustained remission without relapse throughout the follow-up period. The remaining patient, who underwent transplantation in CRi, died from post-transplant complications on day 103. These observations suggest that MVA may serve as a potential bridge to allo-HSCT in selected patients, although the small number of transplanted patients precludes conclusions regarding post-transplant benefit. Further investigation is warranted to determine whether integration of MVA with transplantation strategies may improve long-term outcomes in this setting.

The activity observed in this study, particularly the responses seen in patients previously treated with venetoclax, is biologically plausible in light of emerging preclinical evidence. Venetoclax resistance in AML has been linked to leukaemia stem-cell metabolic adaptation, altered mitochondrial oxidative metabolism, RAS/MAPK-driven anti-apoptotic rewiring and mitochondrial calcium–dependent metabolic states (18, 33, 37–39). In this context, mitoxantrone has been reported in preclinical models to perturb mitochondrial calcium–dependent metabolism and impair venetoclax-resistant LSC function (18). Together with venetoclax-mediated BCL-2 inhibition and the epigenetic effects of azacitidine, these complementary mechanisms provide a biological rationale for combining liposomal mitoxantrone with venetoclax and azacitidine (18, 37, 44–46). The inclusion of patients with early induction failure reflects real-world practice, in which MVA was used as an early salvage strategy after inadequate response to one course of induction therapy to preserve the opportunity for subsequent consolidation or allo-HSCT. Although this may have contributed to the relatively high remission rate, sensitivity analysis excluding these patients showed a consistent efficacy signal. Overall, MVA demonstrated preliminary but clinically meaningful activity in this small real-world cohort, including patients previously treated with venetoclax, with an expected risk of myelosuppression and infection. The observed responses and the potential role of MVA as a bridge to allo-HSCT support further prospective evaluation to confirm efficacy, define the optimal target population and identify molecular or MRD-based predictors of benefit. The main limitations of this study are its retrospective single-centre design, small and heterogeneous cohort, lack of a comparator, and non-standardised post-MVA treatment and serial MRD assessment. Future expansion into prospective, multicentre studies, and ideally randomised controlled trials, will be needed to more comprehensively evaluate the efficacy and safety of this combination regimen.

## Data Availability

The raw data supporting the conclusions of this article will be made available by the authors, without undue reservation.
